# Progress in Establishing Common Standards for Exchanging Proteomics Data: The Second Meeting of the HUPO Proteomics Standards Initiative

**DOI:** 10.1002/cfg.279

**Published:** 2003-04

**Authors:** Sandra Orchard, Paul Kersey, Weimin Zhu, Luisa Montecchi-Palazzi, Henning Hermjakob, Rolf Apweiler

**Affiliations:** 1 EMBL Outstation European Bioinformatics Institute Wellcome Trust Genome Campus Hinxton, Cambridge UK; 2 University of Rome ‘Tor Vergata’ Rome Italy

## Abstract

The Proteomics Standards Initiative (PSI) aims to define community standards for data representation in proteomics and to facilitate data comparison, exchange and verification. Rapid progress has been made in the development of common standards for data exchange in the fields of both mass spectrometry and protein–protein interactions since the first PSI meeting [1]. Both hardware and software manufacturers have agreed to work to ensure that a proteomics-specific extension is created for the emerging ASTM mass spectrometry standard and the data model for a proteomics experiment has advanced significantly. The Protein–Protein Interactions (PPI) group expects to publish the Level 1 PSI data exchange format for protein–protein interactions by early summer this year, and discussion as to the additional content of Level 2 has been initiated.


**Comparative and Functional Genomics**
*Comp Funct Genom* 2003; **4**: 203–206.
Published online 1 April 2003 in Wiley InterScience (www.interscience.wiley.com). **DOI:** 10.1002/cfg.279

**Feature**

### Meeting Review: Progress in Establishing Common Standards for Exchanging Proteomics Data: the second meeting of the HUPO Proteomics Standards Initiative

Hinxton, Cambridge, UK, 22–24 January 2003

Sandra Orchard[1], Paul Kersey[1], Weimin Zhu[1], Luisa Montecchi-Palazzi[2], Henning Hermjakob[1]* and Rolf Apweiler[1]

[1] *EMBL Outstation, European Bioinformatics Institute, Wellcome Trust Genome Campus, Hinxton, Cambridge, UK*
[2] *University of Rome 'Tor Vergata', Rome, Italy*

**Correspondence to: Henning Hermjakob, EMBL Outstation, European Bioinformatics Institute, Wellcome Trust Genome Campus, Hinxton, Cambridge, UK.*
*E-mail: henning.hermjakob@ebi.ac.uk*

Received: 3 February 2003
Revised: 6 February 2003
Accepted: 6 February 2003

### Abstract

**The Proteomics Standards Initiative (PSI) aims to define community standards for data representation in proteomics and to facilitate data comparison, exchange and verification. Rapid progress has been made in the development of common standards for data exchange in the fields of both mass spectrometry and protein–protein interactions since the first PSI meeting [1]. Both hardware and software manufacturers have agreed to work to ensure that a proteomics-specific extension is created for the emerging ASTM mass spectrometry standard and the data model for a proteomics experiment has advanced significantly. The Protein–Protein Interactions (PPI) group expects to publish the Level 1 PSI data exchange format for protein–protein interactions by early summer this year, and discussion as to the additional content of Level 2 has been initiated. Copyright © 2003 John Wiley & Sons, Ltd.**

**Keywords:** **proteomics; spectrometry; protein–protein interactions; standards**

### Introduction

The inaugural meeting of the PSI in October 2002 brought together representatives from the database producer, user and software producer communities who were seen as essential in establishing and maintaining standards in the fields of mass spectrometry and PPIs. The second PSI meeting focused on consolidating and extending both the achievements of the first meeting and work undertaken in the interim period. In addition to this, a wider discussion was initiated on the more global needs of the community. A requirement for standards to allow the storage and exchange of the entire range of proteomics data has been fed back to the PSI over the past few months and there was some debate as to how this could best be addressed.

### Developing a global proteomics standard

One example of such a system is PEDRo, currently under development at the University of Manchester [2]. PEDRo has been designed to encode laboratory produced data, producing an XML-based PEML (proteomics experiment mark-up language) file for local storage or submission to a repository and allows the storage of sample origin and processing information, mass spectrometry data and the results of *in silico* analysis. A related system, YPRC-PDC, was then described by Sangyun

Copyright © 2003 John Wiley & Sons, Ltd.

Cho (Yonsei University, Korea), which has been designed to systematically organize, store and analyse proteomic data. It can act as a repository of data in various formats and has a dynamic user report system. Finally, Martin Blüggel (Protagen AG, Dortmund, Germany) described the difficulties in handling readouts from several different analytical machines. The company have developed a series of algorithms to enable high-throughput data to be collected, compared and fed into a single data management system. The scores are boosted by meta-scoring techniques that rely on known contaminants and internal calibrants. The importance of involving instrument manufacturers from an early stage in any attempt to establish a common standard was heavily stressed throughout the meeting. A decision was taken at this point to use an existing effort, PEDRo, as a starting point for system design, rather than beginning anew.

The meeting then divided into two sessions to concentrate on the more immediate goals of developing standards for mass spectrometry and PPIs.

### Mass spectrometry group

The mass spectrometry group, chaired by Weimin Zhu (EBI), heard a report of a preliminary meeting that had been held between lead players in the first PSI meeting and hardware (Micromass, Bruker and Ciphergen) and software (Water and Mascot) vendors. The vendors had expressed a strong willingness to establish and support a standard representation for proteomics-related mass spectrometry data. Randall Julian (Eli Lilly), the chair of the ASTM (American Society for Tests and Measurements) committee E01.25, which is responsible for an existing mass spectrometry standard, presented this to both the preliminary meeting and the open session. The E01.25 standard has strong vendor support, but its scope does not cover all types of mass spectrometry experiments, and it is implemented in netCDF, which is not a highly descriptive format. Recently two other putative standards, SpectroML and GAML, have emerged, both using XML representations: and a second ASTM committee, E13, is looking at expanding their scope to create a general standard for mass spectrometry to replace the E01.25 standard. However, all existing standards focus on the direct output produced by an instrument and not on its subsequent extension to proteomics.

In general discussions, it was decided to define a standard representation for annotated peak lists (i.e. peak lists plus peptide and protein identifications) and to insert such a representation into the emerging standard for raw mass spectrometry data. Randall Julian agreed to steer a collaborative sharing of the XML schemas already developed by participating vendors, with the intention of reporting back to the PSI within 3 months. PSI will contribute to the process by providing a specification of what information researchers in the field of proteomics would find useful to have captured within such a standard.

### Mass spectrometry and proteomics

The minimum requirements for a possible repository of spectrometric data were discussed. To maximize the interpretability of the results, the full biological context of an experiment, as described in a paper, would need to be deposited; to maximize reproducibility, the full details of the experiment would need to be captured down to the instrument level. However, such a system would be too complex to be practical, and compromises will be made to achieve a practicable system.

Using the prototypic PEDRo data model (as defined in PEML) as a concrete starting point for working on the definition of data structures to hold information on mass spectrometry experiments, the group designed a flexible structure for proteomics experiments, with both separation and analytical phases, to support configurable workflows. The group also developed detailed models for many particular separation techniques. Discussions took place on clearly defining the many different levels at which data is produced or interpreted during a mass spectrometry experiment, and focused in particular on the key question of the representation of peak lists.

### Future developments

The working groups will continue to refine and formalize their data models and hope to collaborate with MGED in developing common guidelines for experimental description. Hardware and software manufacturers have committed to work to ensure

Copyright © 2003 John Wiley & Sons, Ltd.

that a proteomics-specific extension is created for the emerging ASTM mass spectrometry standard. The aim is for a draft specification to be presented to the ASMS (American Society for Mass Spectrometry) meeting in June 2003.

### Protein–protein interactions group

The session opened with a number of presentations describing the analysis of existing PPI data (Michael Lappe, EBI, and Christian von Mering, EMBL) and detailing a number of both PPI and pathway databases (Yves Deville, ULB) that are interested in utilizing the protein interaction data exchange format. Gary Bader described the work of BioPAX, a group formed by a number of databases storing and displaying data on biological pathways (signalling and metabolic) that is also looking for a common format with which to exchange data. BioPAX hope to integrate the standards developed by a number of related projects, such as the PPI data exchange format and chemical markup language (CML) to describe small molecules, into their model to ensure that data exchange will remain possible across the widest possible spread of databases.

### The PSI PPI data exchange format

As previously described [1], the PSI data exchange format is multi-levelled, with Level 1 designed to fulfil basic requirements and be suitable for rapid implementation. The current status of the Level 1 XML model produced as a result of discussions at the first PSI meeting was described by Henning Hermjakob (EBI). The model was then extensively reworked during the open session of the meeting. In addition to the structural changes, researchers also now have the option of ascribing confidence levels to the data at various points throughout the data entry process, dating the entry and adding free comments in appropriate places. Wherever possible, the possible values of attributes in the data model are defined by controlled vocabularies (Figure 1). A number of controlled vocabularies originally developed by Luisa Montecchi (University of Rome) for the IntAct project will be made available as part of the PPI data exchange format. A procedure to add new terms was discussed, which will be implemented before the schema is released.

It is intended to publish the data exchange format and accompanying controlled vocabularies, documentation, examples and use-cases in early summer 2003. A number of the databases represented at the

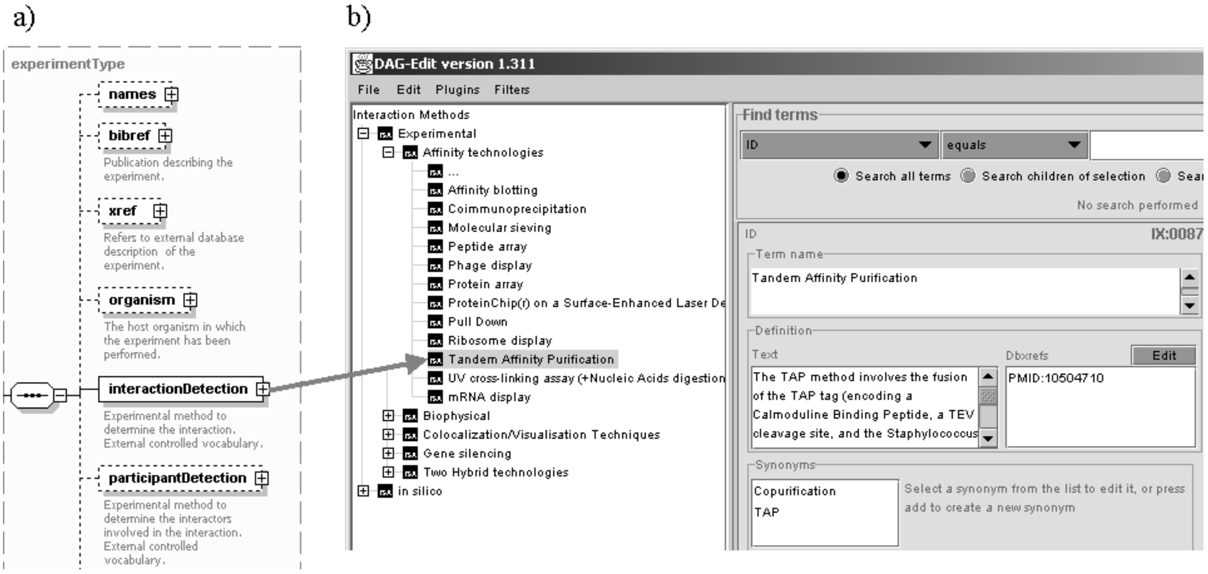

**Figure 1.** (a) A detail of the PSI XML schema for protein interaction data, the description of the experimental parameters. Wherever possible, attributes in the data model are controlled by external controlled vocabularies, which are represented in GO format. (b) An extract of the controlled vocabulary for experimental methods; the arrow illustrates the choice of a value from the controlled vocabulary. The XML schema representation has been generated by XMLSpy 5.3, the representation of the controlled vocabulary by DAG-Edit 1.311

Copyright © 2003 John Wiley & Sons, Ltd. 

meeting, e.g. BIND, DIP, MINT, IntAct and Hybrigenics, intend to offer their publicly available data in this format during 2003, and confirmation from a number of other PPI databases is currently awaited.

### Future developments

Additional features, which will be implemented at Level 2, were briefly discussed during the meeting but it was decided it would not be appropriate to commence this work until Level 1 had been published. The progress of Level 1 and the development of Level 2 will be addressed at the next PSI meeting at the 2nd HUPO congress in Montreal, in October 2003.

### Conclusions

Concrete progress has been made since the initial PSI meeting in October 2002. The mass spectrometry group have aligned themselves with existing efforts to standardize instrumentation, and manufacturers have agreed to support the needs of the proteomics community. Advances have also been made in defining the working model to describe a proteomics experiment. The PPI group are ready to publish Level 1 of the PPI data exchange format by early summer 2003 and the infrastructure to support this initiative is already largely in place.

As previously stated, all such efforts require support from the user community and the PSI is actively seeking input and advice from all quarters. Anyone wishing to become involved is invited to visit **http://psidev.sf.net**, to participate in the discussion groups listed, and to contribute to the further development of community standards for proteomics data.

### Acknowledgements

Supported by European Community Contract No. QLRI-CT-2001-00015 for 'TEMBLOR' under the specific RTD programme 'Quality of Life and Management of Living Resources'.

### Related websites

BIND, **http://bind.ca/**
BioPAX, **http://www.biopax.org**
DIP, **http://dip.doe-mbi.ucla.edu/**
Hybrigenics, **http://www.hybrigenics.fr**
MINT, **http://cbm.bio.uniroma2.it/mint/**
MGED, **http://www.mged.org**
PEDRo, **http://pedro.man.ac.uk/**
PSI, **http://psidev.sf.net/**

### References

1. Orchard S, Kersey P, Hermjakob H, Apweiler R. 2003. Meeting Review: the HUPO Proteomics Standards Initiative meeting: towards common standards for exchanging proteomics data. *Comp Funct Genom* **4**(1): 16–17.
2. Taylor CF, Paton NW, Garwood KL, *et al*. 2003. A systematic approach to modelling, capturing and disseminating proteomics experimental data. *Nature Biotechnol* **21**: 247–254.

Copyright © 2003 John Wiley & Sons, Ltd.
